# *Shank3* deficiency elicits autistic-like behaviors by activating p38α in hypothalamic AgRP neurons

**DOI:** 10.1186/s13229-024-00595-4

**Published:** 2024-04-03

**Authors:** Shanshan Wu, Jing Wang, Zicheng Zhang, Xinchen Jin, Yang Xu, Youwen Si, Yixiao Liang, Yueping Ge, Huidong Zhan, Li peng, Wenkai Bi, Dandan Luo, Mengzhu Li, Bo Meng, Qingbo Guan, Jiajun Zhao, Ling Gao, Zhao He

**Affiliations:** 1 Department of Endocrinology, Shandong Provincial Hospital & Medical Integration, and Practice Center, Shandong University, Jinan, Shandong 250021 China; 2 Key Laboratory of Endocrine Glucose & Lipids Metabolism and Brain Aging, Ministry of Education, Shandong Key Laboratory of Endocrinology and Lipid Metabolism, Shandong Institute of Endocrine and Metabolic Diseases, Shandong Clinical Research Center of Diabetes and Metabolic Diseases, Shandong Prevention and Control Engineering Laboratory of Endocrine and Metabolic Diseases, Shandong Provincial Hospital Affiliated to Shandong First Medical University, Jinan, Shandong 250021 China; 3 Key Laboratory of Cardiovascular Remodeling and Function Research, Chinese Ministry of Education, Chinese National Health Commission and Chinese Academy of Medical Sciences, The State and Shandong Province Joint Key Laboratory of Translational Cardiovascular Medicine, Department of Cardiology, Qilu Hospital, Cheeloo College of Medicine, Shandong University, Jinan, Shandong 250021 China; 4 School of Modern Posts, Nanjing University of Posts and Telecommunications, Nanjing, Jiangsu 210009 China; 5 Advanced Medical Research Institute, Cheeloo College of Medicine, Shandong University, Jinan, Shandong 250012 China; 6 Key Laboratory of Brain Functional Genomics, Ministry of Education, School of Life Sciences，East China Normal University, Shanghai, 200062 China; 7 Department of Pharmacology and Chemical Biology, Department of Neurology, Emory University, Atlanta, GA 30322 USA; 8 Cheeloo College of Medicine, Shandong Provincial Hospital, Shandong University, 544 Jingsi Road, Jinan, Shandong 250021 China

**Keywords:** Autism, p38α, SHANK3, AgRP, Stereotypic behavior, Sociability

## Abstract

**Background:**

SH3 and multiple ankyrin repeat domains protein 3 (SHANK3) monogenic mutations or deficiency leads to excessive stereotypic behavior and impaired sociability, which frequently occur in autism cases. To date, the underlying mechanisms by which *Shank3* mutation or deletion causes autism and the part of the brain in which *Shank3* mutation leads to the autistic phenotypes are understudied. The hypothalamus is associated with stereotypic behavior and sociability. p38α, a mediator of inflammatory responses in the brain, has been postulated as a potential gene for certain cases of autism occurrence. However, it is unclear whether hypothalamus and p38α are involved in the development of autism caused by *Shank3* mutations or deficiency.

**Methods:**

Kyoto Encyclopedia of Genes and Genomes (KEGG) pathway analysis and immunoblotting were used to assess alternated signaling pathways in the hypothalamus of *Shank3* knockout (*Shank3*^*−/−*^) mice. Home-Cage real-time monitoring test was performed to record stereotypic behavior and three-chamber test was used to monitor the sociability of mice. Adeno-associated viruses 9 (AAV9) were used to express p38α in the arcuate nucleus (ARC) or agouti-related peptide (AgRP) neurons. D176A and F327S mutations expressed constitutively active p38α. T180A and Y182F mutations expressed inactive p38α.

**Results:**

We found that *Shank3* controls stereotypic behavior and sociability by regulating p38α activity in AgRP neurons. Phosphorylated p38 level in hypothalamus is significantly enhanced in *Shank3*^*−/−*^ mice. Consistently, overexpression of p38α in ARC or AgRP neurons elicits excessive stereotypic behavior and impairs sociability in wild-type (WT) mice. Notably, activated p38α in AgRP neurons increases stereotypic behavior and impairs sociability. Conversely, inactivated p38α in AgRP neurons significantly ameliorates autistic behaviors of *Shank3*^*−/−*^ mice. In contrast, activated p38α in pro-opiomelanocortin (POMC) neurons does not affect stereotypic behavior and sociability in mice.

**Limitations:**

We demonstrated that SHANK3 regulates the phosphorylated p38 level in the hypothalamus and inactivated p38α in AgRP neurons significantly ameliorates autistic behaviors of *Shank3*^*−/−*^ mice. However, we did not clarify the biochemical mechanism of SHANK3 inhibiting p38α in AgRP neurons.

**Conclusions:**

These results demonstrate that the *Shank3* deficiency caused autistic-like behaviors by activating p38α signaling in AgRP neurons, suggesting that p38α signaling in AgRP neurons is a potential therapeutic target for *Shank3* mutant-related autism.

**Supplementary Information:**

The online version contains supplementary material available at 10.1186/s13229-024-00595-4.

## Background

Autism spectrum disorders (ASD) are associated with severe stereotypical behavior and social impairments [1] and primarily caused by gene mutations. *Shank3* mutation or deficiency frequently occurs in autism cases [2–4]. *Shank3* deficiency impairs the structure and function of synapses, which affects the neural networks that are vital for individuals, thereby leading to ASD [4–6]. Indeed, restored SHANK3 expression in animal models reverses some of the deficits in synaptic function and autistic-like behaviors [3]. Notably, *Shank3* is a core excitatory postsynaptic protein that regulates synaptic function in multiple brain regions including the medial prefrontal cortex (mPFC), striatum, and hippocampus [7–9]. *Shank3*-altered mice and children with autism often have eating disorders [3, 10, 11], and the center of feeding regulation is hypothalamus [12]. However, the role of the hypothalamus in *Shank3* deletion or mutation-caused autism is still poorly understood.

AgRP neurons are a class of neurons located in the arcuate nucleus of the hypothalamus and primarily recognized for controlling feeding and energy metabolism [13]. Substantial literatures have shown that AgRP neurons are implicated in regulating core symptoms of autism beyond feeding. For example, activation of AgRP neurons in the absence of food drives stereotypic behaviors [14]. Consistently, the activation of melanocortin 4 receptor (MC4R), a downstream molecular of AgRP, induces excessive stereotypic behaviors [15]. On the other hand, AgRP neurons also participate in regulating social behaviors due to their critical role in controlling the structure and function of the mPFC [16, 17]. Notably, AgRP neurons are 5-hydroxytryptamine (5-HT) receptor-positive neurons, which are strongly associated with autism [18–20]. Thus, whether AgRP neurons are associated with the *Shank3* deficiency-caused autism is still unknown.

Previous studies have shown that 5-HT transporter (SERT) mutations cause alterations in SHANK3 signaling pathway, and inhibition of p38α cures autistic-like behaviors in SERT mutant mice [19, 21]. p38α, a member of the mitogen-activated protein kinase (MAPK) family, is involved in various cellular processes, including inflammation, cell differentiation, and response to stress [22]. In particular, p38α is a key mediator of inflammatory responses in the brain, which has been postulated as a potential risk factor for certain cases of autism occurrence [23–25]. Indeed, alterations in the upstream or downstream signaling of p38α affect autistic symptoms [26–28]. Of note, p38α in 5-HT neurons drives autistic-like phenotypes directly in the ASD model [18, 19]. However, it is still unknown whether and how p38α plays a crucial role in ASD development due to its wide expression in multiple tissues and differential functions [29–31].

Here, we demonstrate that hypothalamic p38 is activated in *Shank3* deficiency mice. Overexpression of p38α in ARC or activating p38α in hypothalamic AgRP neurons elicits autistic behaviors. Conversely, inactivating p38α in AgRP neurons ameliorates autistic behaviors in *Shank3*^−/−^ mice. Our results demonstrate that p38α signaling in AgRP neurons is one of the SHANK3 downstream pathways in controlling autistic behaviors.

## Methods

### Animals

WT (C57BL/6J) mice were purchased from Charles River Laboratory (219, Beijing, China), *Shank3*^*−/−*^ mice (017688), BTBR mice (002282), *Agrp-Cre* mice (012899), *POMC-Cre* mice (010714) were purchased from Jax lab (USA), *p38α*^*D176A−F327S*^ and *p38α*^*T180A−Y182F*^ flox knock-in mice were generated by GemPharmatech (Nanjing, China), *p38α*^*flox/flox*^ knock-in mice were kindly provided by Lijian Hui [32], and Tomato-reporter mice were purchased from GemPharmatech (T002249, Nanjing, China). All animals were kept under standard conditions with temperature (22 ± 1 °C) and humidity (~ 40%) in a 12 h light/12 hours dark cycle, with free access to food and water. Mice were used for experiments at 10–12 weeks of age. All animal experiments were permitted by the Institutional Animal Care and Use Committee at Shandong Provincial Hospital and complied with the China National Regulations on the Administration of Experimental or Laboratory Animals (No.2, 20,170,301, SSTC, China), and the ARRIVE guidelines or the U.K. Animals (Scientific Procedures) Act, 1986.

### Genotyping

Genotyping was conducted as previously report [33]. In brief, 2 mm mouse tails were harvested at 3 weeks of age. Primers are as follow: For *p38α*^*flox/flox*^: Forward (F): GTCCCGAGAGTTCCTGCCTC, Reverse (R): CGCGAGAACAGCTCCAAGGAG; For *Agrp-Cre* and *POMC-Cre*: F: GCAACGAGTGATGAGGTTCGCAAG, R: CTAAGTGCCTTCTCTACACCTGCGG; For T180A-Y182F and D176A-F327S mutation: mutant fragment: F: CCTCCTCTCCTGACTACTCCCAGTC, R: CAAGTCAGGATTTAAGACACCC, WT fragment: F: CTCTCACCCTTCATTCAATGCAG, R: GTCACTTTCAGAAAGAGGGCACC; For *Shank3*^−/−^: mutant fragment: F: GAGACTGATCAGCGCAGTTG, R: GCTATACGAAGTTATGTCGACTAGG, WT fragment: F: GAGACTGATCAGCGCAGTTG, R: TGACATAATCGCTGGCAAAG.

### Viruses information and stereotaxic surgeries

The CMV-EGFP-P2A-Cre-WPRE (*AAV9-CMV-Cre*, 1E + 12v.g/ml), CMV-bGlobin-FLEX-MCS-EGFP-WPRE-hGH-polyA (*AAV9-con*, 1E + 12v.g/ml), CMV-bGlobin-FLEX-Mapk14-EGFP-WPRE-hGH-polyA (*AAV9-p38α*^*flox/flox*^, 1E + 12v.g/ml), AgRP-Cre-SV40-polyA (*AAV9-Agrp-Cre*, 1E + 12v.g/ml), and AgRP-SV40-polyA (*AAV9-Agrp-con*, 1E + 12v.g/ml) were generated and purified by Genechem Co., LTD. (Shanghai, China). The full names of the elements in the virus vectors are listed in the section “abbreviations”. For the overexpression of p38α in ARC, WT mice were injected with a mix (0.5ul/side) of *AAV9-p38α*^*flox/flox*^ or *AAV9-con* and *AAV9-CMV-Cre* bilaterally into ARC (bregma as the reference point, anteroposterior: −1.7 mm, mediolateral: ±0.2 mm, dorsoventral: −5.8 mm). For the overexpression of p38α in AgRP neurons, *Agrp-Cre* mice were injected with *AAV9-p38α*^*flox/flox*^ or *AAV9-con* bilaterally into ARC (0.5 µl/side). For the generation of *p38α*^*AgRP−KD*^ mice, *p38α*^*flox/flox*^ mice were injected with *AAV9-Agrp-Cre* or *AAV9-Agrp-con* bilaterally into ARC (0.5 µl /side). Viruses were stereotaxically infused into ARC as previously report [31]. Mice with injection sites out of the ARC were excluded, and only those animals with injection sites in the ARC were included in the study.

### Home-cage monitoring test

Mice were kept in individual cages for behavior monitoring, with a 12 h light/12 hours dark cycle and an ambient temperature of 24 ± 2 °C in a silent room. Mice were acclimated to the cages for 24 h before starting records, and their behaviors were recorded for 24 h from 7 postmeridiem to 7 postmeridiem the next day. Fluorescent lights simulate daytime and lights off simulate night, without disrupting the normal circadian rhythm of mice. Mice were monitored by an infrared camera (Shanghai Vanbi Intelligent Technology Co., Ltd.) mounted horizontally on the side of the cage for 24 h. Video data were analyzed by Tracking Master V3.1.56 software (Shanghai Vanbi Intelligent Technology Co., Ltd.), and behavioral definitions were as described previously [34]. Tracking Master V3.1.56 software monitored parameters as follows: contour erosion (2 pixels), contour expansion (2 pixels), animal size (500-10000 pixels), motionless (0–4 cm/s), active ( > = 4 cm/s), slow active (4–10 cm/s), fast active ( > = 20 cm/s), move between two frames (1-10000 pixels) [35].

### Three chamber tests

The test was performed as previously described [36]. Before the three-chamber test, each mouse was acclimatized in three chambers for five minutes, and three hours later, the first and second phases of the experiment were conducted. In the first stage, an unfamiliar mouse (stranger 1) was placed in chamber (1) The mouse was placed in the middle chamber and allowed to travel freely in the three chambers. The trajectory of mice was recorded by an automatic video tracking system (Tracking Master V3.0, Shanghai Vanbi Intelligent Technology Co., Ltd.) for ten minutes and the time spent in each chamber was counted. In the second phase of the experiment, the mouse in chamber 1 from the first phase was maintained, while a new unfamiliar mouse (stranger 2) was placed in chamber (2) Again, the trajectories of mice were recorded for ten minutes, and the time spent in each chamber was counted.

### Transcriptomic analysis

Transcriptomic data of primary neurons transduced with *Shank3* short hairpin ribonucleic acid (shRNA, GSE47150) and hypothalamus in *Shank3*-overexpressing (*Shank3*^*TG*^) mice (GSE120609) were downloaded from https://www.ncbi.nlm.nih.gov. Data were imported into the R-package ‘limma’ for differential gene screening. Fold change was set at 1.2. Then, the KEGG pathways were enriched by ‘enrichKEGG’ in the R-package ‘clusterProfiler’. These pathways were sorted by count and selected the first 20 pathways for presentation.

### Immunoblotting

Hypothalamus tissues were lysed in Radio Immunoprecipitation Assay (RIPA) lysis buffer (Shenergy Biocolor Bioscience & Technology Co.) containing protease inhibitors and phosphatase inhibitors. Tissue lysates were immunoblotted with antibodies: anti-p38 (1:1000, 9212, Cell Signaling Technology), anti-phospho-p38 (p-p38; 1:1000, 4511, Cell Signaling Technology), anti-phospho-extracellular signal-regulated kinase (p-ERK; 1:1000, 4376, Cell Signaling Technology), anti-ERK (1:1000, 4695, Cell Signaling Technology), anti-phospho-c-Jun NH2-terminal kinase (p-JNK; 1:1000, 9255, Cell Signaling Technology), anti-JNK (1:1000, 9252, Cell Signaling Technology), glyceraldehyde-3-phosphate dehydrogenase (GAPDH; 1:7500, 60004-1-Ig, Proteintech), and Tubulin (1:5000, 11224-1-AP, Proteintech). 40 µg protein in each tissue lysate was loaded in the gel. The signals were detected by Amersham Imager 680. Protein band intensities were quantified using ImageJ (National Institutes of Health).

#### Immunofluorescence staining

Brains were harvested and maintained in 4% paraformaldehyde overnight at 4 °C. The following day, brains were placed in 10 ml of 30% sucrose overnight. Brains were sectioned (40 μm, Leica SM 200 R, Leica Biosystems, Nussloch, Germany) and stored at − 20 °C in freezing medium (30% ethylene glycol, 25% glycerol in phosphate-buffered saline (137 mM NaCl, 2.7 mM KCl, 10 mM Na2HPO4, 1.8 mM KH2PO4, pH 7.4) before analysis. Sections were stained with anti-p38α (1:800, AF8691, R&D Systems), anti-p38α (1:800, 4511, cell Signaling Technology, Inc.), and anti-AgRP (1:500, PA5-47831, Invitrogen) antibodies overnight at 4 °C, and then with tetramethylrhodamine isothiocyanate (TRITC) or fluorescein isothiocyanate (FITC) labeled secondary antibodies (donkey anti-rabbit or donkey anti-goat) for 1 h at room temperature. Sections were mounted with 4’,6-diamidino-2-phenylindole (DAPI, Thermo Fisher Scientific Inc.) and coverslipped. Immunofluorescence sections were observed by a Leica SP5 confocal microscope (Leica Microsystems GmbH, Mannheim, Germany, 405 nm laser for DAPI, 488 nm laser for FITC and GFP, and 561 nm laser for TRITC and tomato), and images were processed and assembled using Photoshop 2022 (Adobe, Inc.).

### Lipopolysaccharide (LPS) challenge assay

LPS was used to activate hypothalamic p38α [37, 38], the mouse was intraperitoneally injected with 1 mg/kg LPS for 30 min, and then the hypothalamus was collected for immunofluorescence staining.

## Results

### Hypothalamic p38 is activated in ASD mouse model

**Fig. 1:**
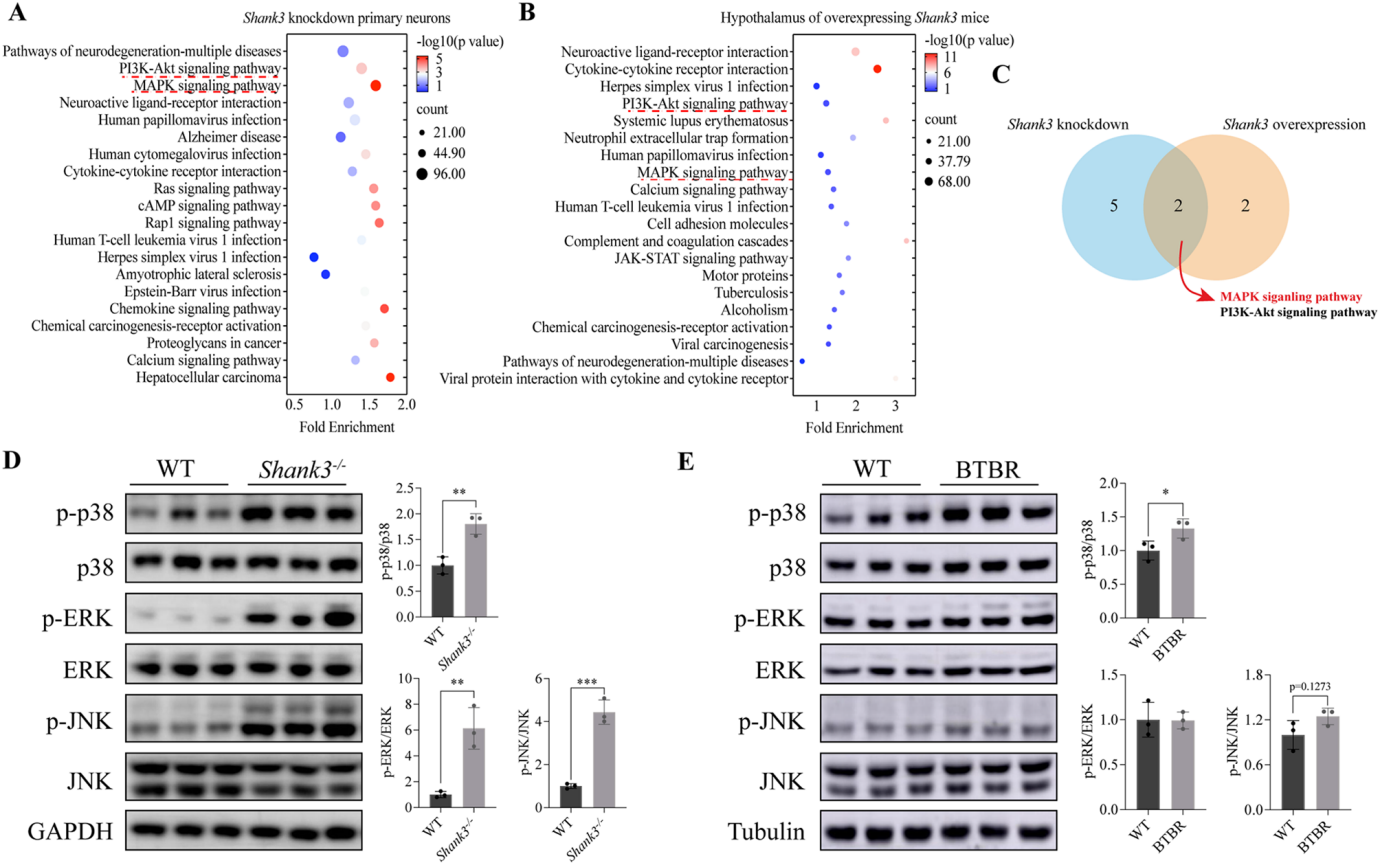
Phosphorylated p38 level in the hypothalamus is significantly enhanced in ASD mouse model. **A**. KEGG enrichment analysis of the transcriptome from Shank3 knockdown primary neurons (GSE47150). **B**. KEGG enrichment analysis for the hypothalamus of Shank3TG mice (GSE120609). **C**. Overlapping of signaling pathways of two datasets, the number represents the well-defined pathways, and non-defined pathways were excluded. **D**. Immunoblotting and intensity of phosphorylation of p38, ERK1/2, and JNK in hypothalamus from WT and *Shank3*^*−/−*^ mice (*n* = 3 per group). **E**. Immunoblotting and intensity of phosphorylated p38, ERK1/2, and JNK in hypothalamus from WT, BTBR mice (*n* = 3 per group). Statistical analysis: data were analyzed using unpaired two-tailed Student’s *t*-test (Prism9, GraphPad Software Inc.). Data were represented as Mean ± SD. Significance levels are indicated with *p < 0.05, **p < 0.01, ***p < 0.001

*Shank3* may play a role in the hypothalamus due to the altered food intake in *Shank3* deletion and overexpression mice [3, 10]. Notably, the hypothalamus regulates not only feeding behaviors but also stereotypic behavior and sociability [14, 16, 39], the latter being the typical symptoms of autism [1]. Thus, it raises a possibility that the hypothalamus may be an important brain site in *Shank3*-related autism. To explore whether the hypothalamus is involved in the regulation of *Shank3* deletion-caused autistic-like behaviors, we analyzed the alternations of signaling pathways profiles in *Shank3* knockdown primary cortical neurons (GSE47150) [40] and hypothalamus of mice with overexpressing *Shank3* (GSE120609), respectively (Fig. 1A, B) [41]. Of note, the overlapping of KEGG pathways enrichment from the two datasets showed that the phosphoinositide 3-kinase (PI3K/Akt) signaling pathway and MAPK signaling pathway are significantly altered (Fig. 1A, B, C). Indeed, the PI3K/Akt and MAPK signaling pathways are strongly associated with neurodevelopment in children with autism [18, 19, 42, 43]. Although there is a strong correlation between MAPK and PI3K/Akt signaling pathways and autism [21, 44], we are more interested in whether MAPKs pathway are involved in regulating *Shank3*-associated autism.

Next, we determined the phosphorylation levels of typical MAPKs [45]: p38, ERK1/2, and JNK in the hypothalamus by immunoblotting. *Shank3*^*−/−*^ mice displayed higher levels of p-p38, p-Erk1/2, and p-JNK compared to WT mice (Fig. 1C), indicating an inhibitory effect of SHANK3 on the MAPK pathway. Consistently, substantial literatures have shown that SHANK3 in synaptic components is regulated via signaling cascades p38α [19, 46] and ERK2 [47]. Notably, the level of hypothalamic p-p38α was also higher in BTBR mice (another ASD mouse model) than in WT mice (Fig. 1D). Furthermore, other labs and our previous studies found that the mutation of Shp2, an upstream molecule of p38α, leads to Noonan syndrome, accompanied by obesity and autism symptoms [26, 28, 48]. Inhibition of MAP kinase interacting kinase (MNK1/2), a downstream effector of p38α, restores social behavior in ASD mouse model [27]. Thus, these findings raise a possibility that hypothalamic p38α is a possible downstream molecule of SHANK3 to regulate stereotypic behavior and sociability.

### p38α overexpression in ARC is sufficient to elicit excessive stereotypic behavior and impaired sociability in WT mice

**Fig. 2:**
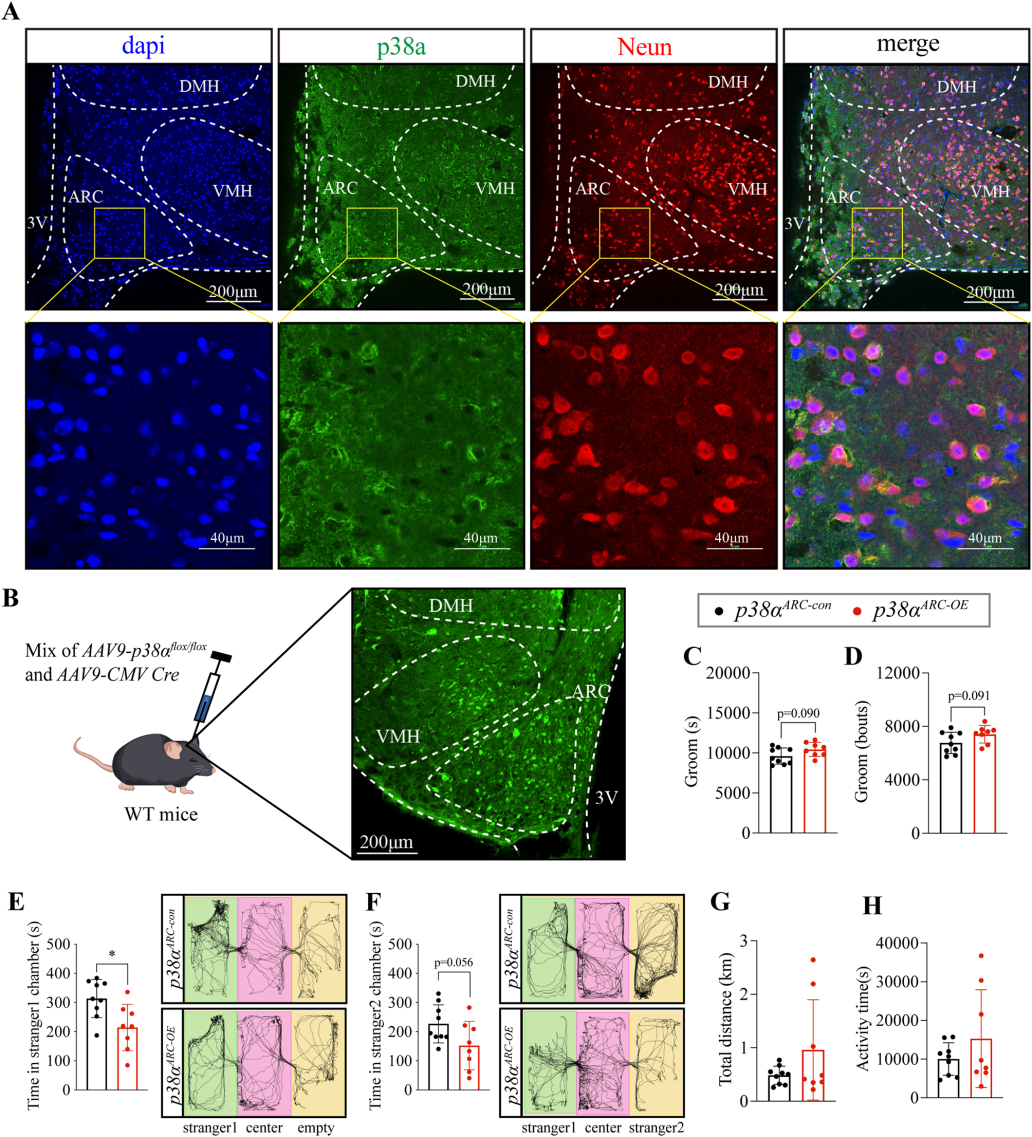
p38α overexpression in ARC is sufficient to elicit excessive stereotypic behavior and impaired sociability in WT mice. **A**. Immunofluorescence staining of DAPI (blue, 405 nm), p38α (green, 488 nm, FITC), and Neun (red, 561 nm, TRITC) in ARC. **B**. Schematic and immunofluorescence image of the *AAV9-p38α*^*flox/flox*^ and *AAV9-Agrp-Cre* mix injection to ARC in WT mice, green fluorescent protein (GFP, 488 nm) represents virus expression. **C** and **D**. Groom time and bouts in 24 h of *p38α*^*ARC−con*^ and *p38α*^*ARC−OE*^ mice. **E**. The first phase of the three-chamber test, the time of *p38α*^*ARC−con*^ and *p38α*^*ARC−OE*^ mice in stranger1 chamber. **F**. The second phase of the three-chamber test, the time of *p38α*^*ARC−con*^ and *p38α*^*ARC−OE*^ mice in stranger2 chamber. **G**. Total distance in 24 h of *p38α*^*ARC−con*^ and *p38α*^*ARC−OE*^ mice. **H**. Activity time in 24 h of *p38α*^*ARC−con*^ and *p38α*^*ARC−OE*^ mice. 10-week-old WT male mice were injected with virus. The injection site is in the ARC area as an inclusion criterion. 12 injected mice in the *p38α*^*ARC−con*^ group, 3 mice were excluded and 9 mice were included in the experiment. 11 injected mice in the *p38α*^*ARC−OE*^ group, 3 mice were excluded and 8 mice were included in the experiment. Home-Cage monitoring test was performed 6 weeks after virus injection, three-chamber test was performed 8 weeks after virus injection. Statistical analysis: data were analyzed using unpaired two-tailed Student’s *t*-test (Prism9, GraphPad Software Inc.). Data were represented as Mean ± SD. Significance levels are indicated with *p < 0.05.

Since ARC in the hypothalamus is a regulatory nucleus for behaviors [14, 16, 39]. Next, we determined the expression of p38α by immunostaining and found that p38α highly expressed in ARC neurons (Fig. 2A). However, whether p38α in ARC neurons is sufficient to regulate stereotypic behavior and sociability is still unknown. To address this issue, we generated *p38α*^*ARC−OE*^ mice, which allows for overexpressing *p38α* in ARC (Fig. 2B). As expected, *p38α*^*ARC−OE*^ mice showed an increased trend in stereotypic behavior (Fig. 2C, D) and impaired sociability (Fig. 2E) compared to control mice, accompanied by an impaired trend of preference for social novelty (Fig. 2F). Total distance and activity time were unchanged (Fig. 2G, H). Thus, these results demonstrate that overexpressing p38α in ARC is sufficient to elicit excessive stereotypic behavior and impaired sociability.

### Overexpression of p38α in AgRP neurons elicits excessive stereotypic behavior and impaired sociability in WT mice

**Fig. 3:**
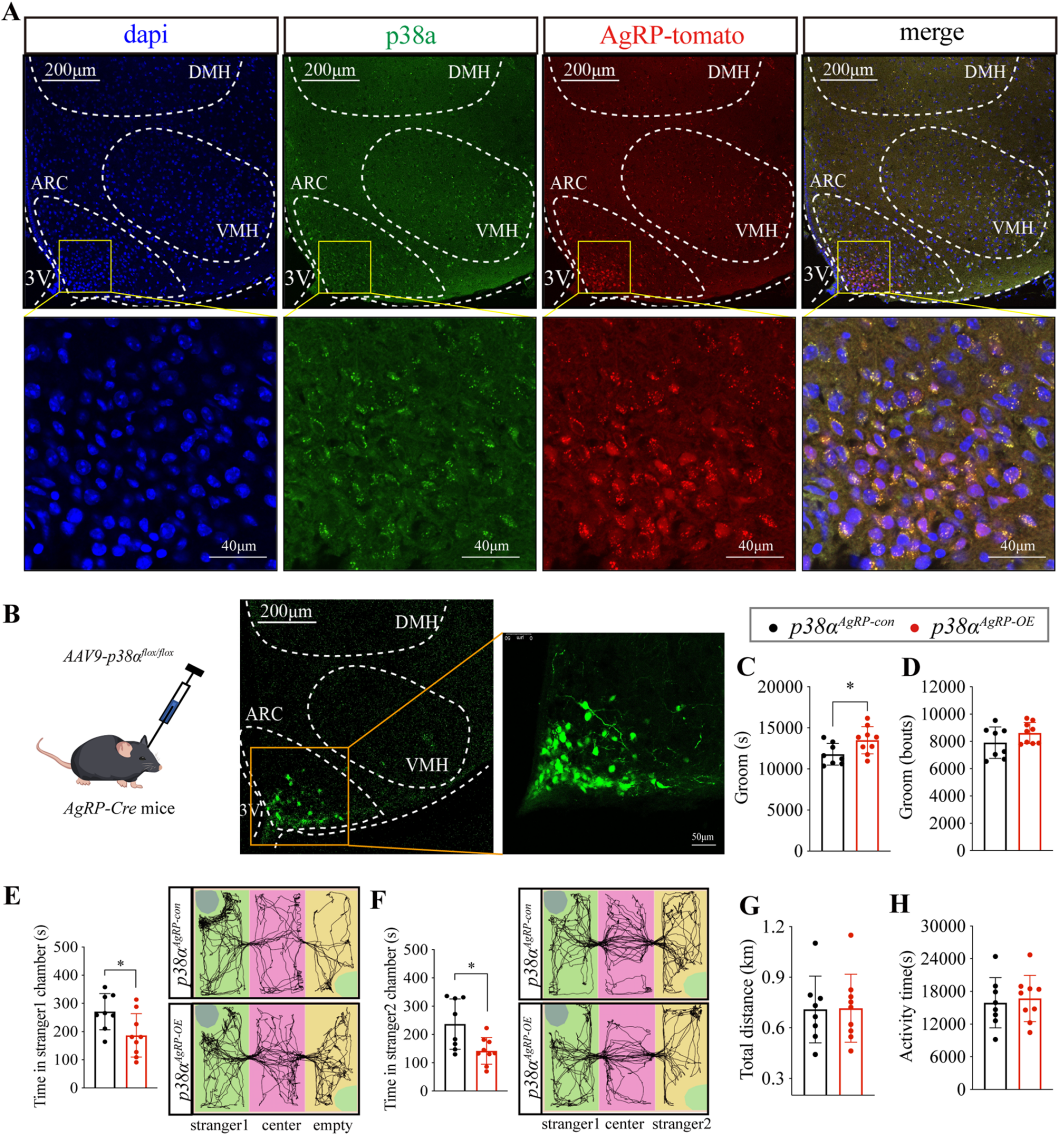
Overexpression of p38α in AgRP neurons elicits excessive stereotypic behavior and impaired sociability in WT mice. **A**. Immunofluorescence staining of DAPI (blue, 405 nm) and p38α (green, 488 nm, FITC) in ARC, AgRP-tomato mice were generated by mating *Agrp-Cre* mouse with Tomato-reporter mouse (red, 561 nm). **B**. Schematic and immunofluorescence image of the *AAV9-p38α*^*flox/flox*^ stereotaxic injection to ARC in *Agrp-Cre* mice, GFP represents virus expression. **C** and **D**, Groom time and bouts in 24 h of *p38α*^*AgRP−con*^ and *p38α*^*AgRP−OE*^ mice. **E**. The first phase of the three-chamber test, the time of *p38α*^*AgRP−con*^ and *p38α*^*AgRP−OE*^ mice in stranger1 chamber. **F**. The second phase of the three-chamber test, the time of *p38α*^*AgRP−con*^ and *p38α*^*ARC−OE*^ mice in the stranger2 chamber. **G**. The total distance in 24 h of *p38α*^*AgRP−con*^ and *p38α*^*AgRP−OE*^ mice. **H**. The activity time in 24 h of *p38α*^*AgRP−con*^ and *p38α*^*AgRP−OE*^ mice. 10-week-old control and *AgRP-Cre* male mice were injected with *AAV9-p38α*^*flox/flox*^. The injection site is in the ARC area as an inclusion criterion. 12 injected mice in the *p38α*^*AgRP−con*^ group, 4 mice were excluded and 8 mice were included in the experiment. 12 injected mice in the *p38α*^*AgRP−OE*^ group, 3 mice were excluded and 9 mice were included in the experiment. Home-Cage monitoring test was performed 4–6 weeks after AAV injection, three-chamber test was performed 8–10 weeks after AAV injection. Statistical analysis: data were analyzed using unpaired two-tailed Student’s *t*-test (Prism9, GraphPad Software Inc.). Data were represented as Mean ± SD. Significance levels are indicated with *p < 0.05.

Previous studies unveiled that AgRP neurons in ARC regulate stereotypic behavior and sociability [14, 16, 39]. Thus, these findings raise a possible regulatory effect of p38α in AgRP neurons on stereotypic behavior and sociability. For this reason, we first examined the expression of p38α in AgRP neurons and found that AgRP neurons highly express p38α (Fig. 3A). Next, we generated *p38*α^*AgRP−OE*^ mice by expressing *AAV9-p38α*^*flox/flox*^ in *Agrp-cre* mice, which allows for *p38α* specifically overexpressing in AgRP neurons (Fig. 3B). *p38*^*AgRP − OE*^ mice showed a significant increase in stereotypic behavior (Fig. 3C, D), sociability and preference for social novelty were dramatically impaired (Fig. 3E, F), accompanied by unaltered total distance and activity time (Fig. 3G, H). Consequently, these results demonstrate that p38α in AgRP neurons is sufficient to elicit excessive stereotypic behavior and impaired sociability.

Next, we determined whether p38α is necessary for stereotypic behavior and sociability. For this, we specifically deleted *p38α* in AgRP neurons (*p38*^*AgRP − KO*^) by *p38*^*flox/flox*^ mice crossed with *Agrp-Cre* mice (Fig. S1A, B). *p38α* deficiency in AgRP neurons did not cause significant changes in stereotypic behavior and sociability (Fig. S1C-H), consistent with the redundant functions of *p38α* isoform [9]. Similarly, the knockdown of *p38α* in AgRP neurons (*p38α*^*AgRP−KD*^) by expressing *AAV9-Cre* in *p38α*^*flox/flox*^ mice did not lead to any changes in stereotypic behavior and sociability (Fig. S2).

### Activated p38α in AgRP neurons elicits excessive stereotypic behavior and impaired sociability in WT mice

**Fig. 4:**
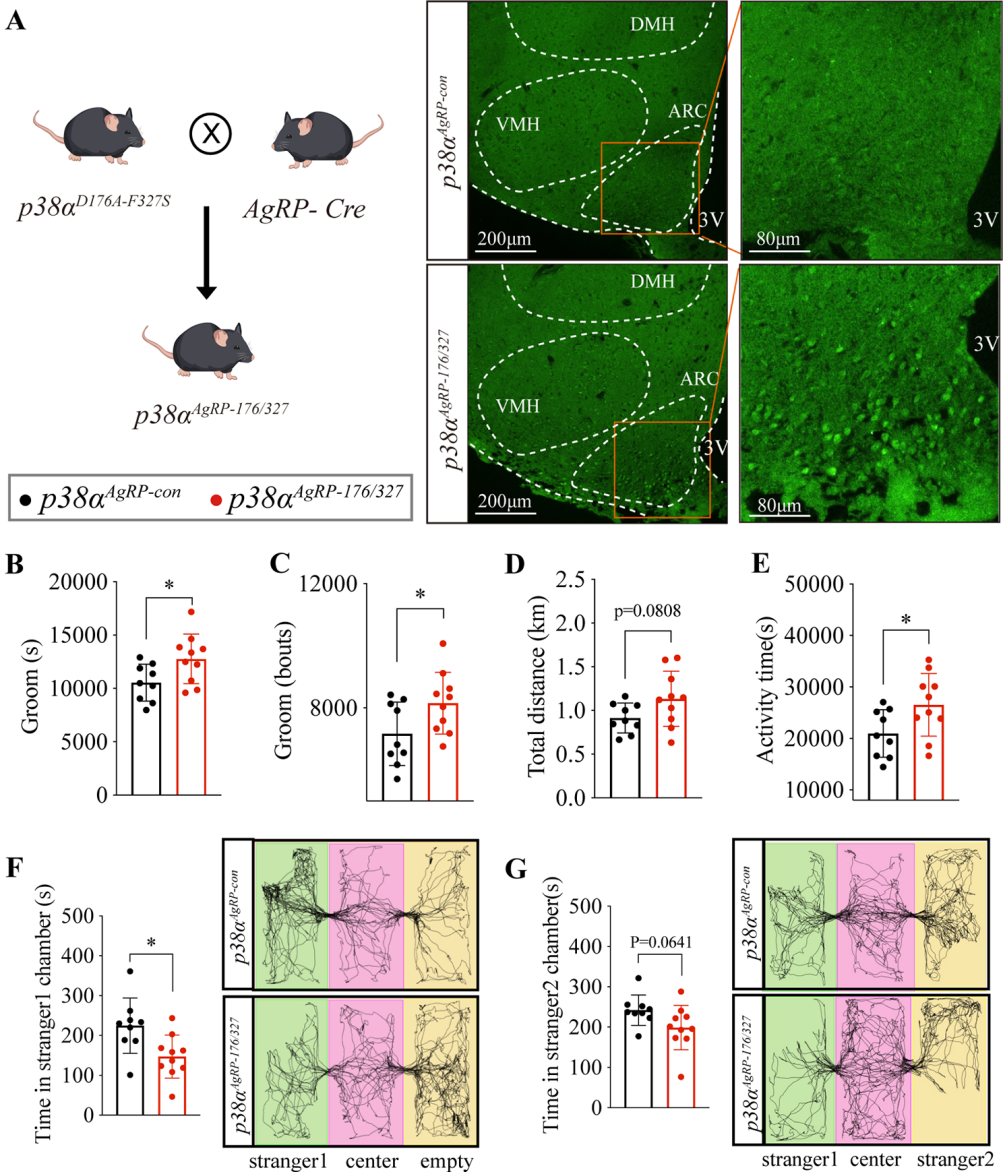
Activated p38α in AgRP neurons elicits excessive stereotypic behavior and impaired sociability in WT mice. **A**. schematic of generating *p38α*^*AgRP-176/327*^ mice and immunofluorescence staining of p-p38α (green, 488 nm, FITC). **B** and **C**, the groom time and bouts in 24 h of *p38α*^*AgRP-con*^ and *p38α*^*AgRP-176/327*^ mice. **D**. the total distance in 24 h of *p38α*^*AgRP-con*^ and *p38α*^*AgRP-176/327*^ mice. **E**. the activity time in 24 h of *p38α*^*AgRP-con*^ and *p38α*^*AgRP-176/327*^ mice. **F**. the first phase of the three-chamber test, the time of *p38α*^*AgRP-con*^ and *p38α*^*AgRP-176/327*^ mice in stranger1 chamber. **G**. the second phase of the three-chamber test, the time of *p38α*^*AgRP-con*^ and *p38α*^*AgRP-176/327*^ mice in stranger2 chamber. *p38α*^*AgRP-con*^ and *p38α*^*AgRP-176/327*^ male mice were used for the experiment, 8 mice in *p38α*^*AgRP-con*^ and 9 mice in *p38α*^*AgRP-176/327*^ group. Mice were subjected to Home-Cage monitoring test at the age of 8–9 weeks, and subjected to three-chamber test at the age of 9–10 weeks. Statistical analysis: data were analyzed using unpaired two-tailed Student’s *t*-test (Prism9, GraphPad Software Inc.). Data were represented as Mean ± SD. Significance levels are indicated with *p < 0.05.

Since the levels of hypothalamic p-p38α were upregulated in *Shank3*^−/−^ mice (Fig. 1C), we next determined whether the activation of p38α is sufficient to regulate stereotypic behavior and sociability. For this, we generated a *p38α*^*AgRP−176/327*^ mouse line by *p38α*^*D176A−F327S*^ flox knock-in mice (Fig. S3A) crossed with *Agrp-Cre* mice, in which D176A and F327S mutations lead to spontaneous and sustained activation of p38α in AgRP neurons [49] (Fig. 4A). Similar to *p38*α^*AgRP−OE*^ mice, *p38α*^*AgRP−176/327*^ mice displayed a significant increase in stereotypic behavior (Fig. 4B, C), slightly elevate total distance, increased activity time (Fig. 4D, E), and impaired sociability (Fig. 4F) accompanied by an impaired trend of preference for social novelty (Fig. 4G). Together, activation of p38α in AgRP neurons causes excessive stereotypic behavior and impaired sociability.

To investigate if the p38α activity in AgRP neurons is necessary for regulating stereotypic behavior and sociability, we generated a *p38α*^*AgRP−180/182*^ mouse line by *p38α*^*T180A−Y182F*^ flox knock-in mice (Fig. S3B) crossed with *Agrp-Cre* mice, in which we specifically inactivated p38α in AgRP neurons by mutating residues both T180A and Y182F in p38α [50] (Fig. S4A, B). Consistent with the results of *p38α* deletion in AgRP neurons, *p38α*^*AgRP−180/182*^ mice displayed an unchanged stereotypic behavior (Fig. S4C, D), similar total distance (Fig. S4E), unaltered activity time (Fig. S4F), and unchanged sociability (Fig. S4G, H) compared to *p38α*^*AgRP−con*^ mice. These results demonstrate that activation of *p38α* in AgRP neurons is not necessary for regulating stereotypic behavior and sociability in WT mice.

### Inactivated p38α in AgRP neurons ameliorates the autistic-like behaviors of *Shank3*^-/-^ mice

**Fig. 5:**
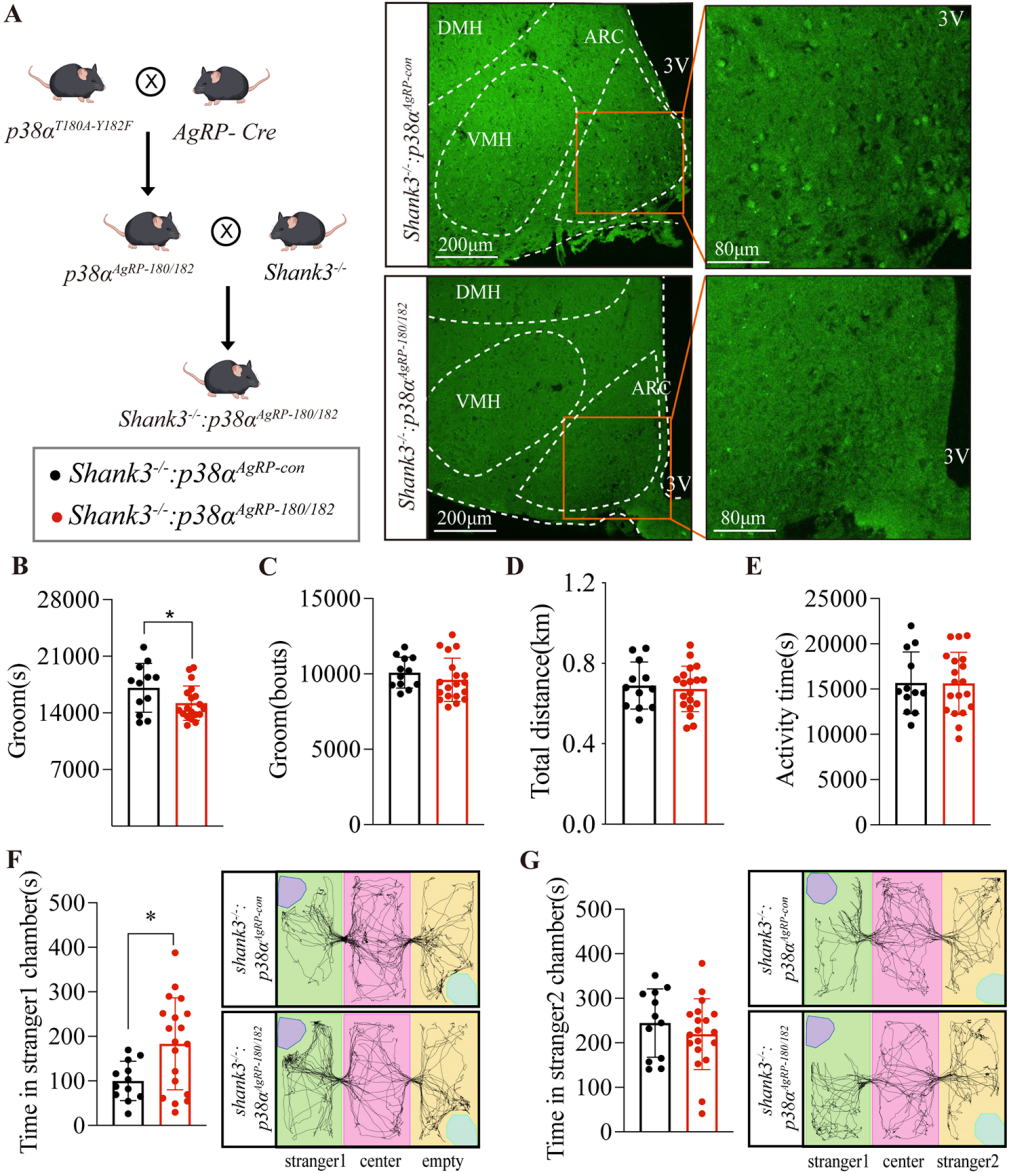
Inactivated p38α in AgRP neurons ameliorates the autistic-like behaviors of *Shank3*^-/-^ mice. **A**. scheme of generating *Shank3*^-/-^:*p38α*^*AgRP-180/182*^ mice and immunofluorescence staining of p-p38α(green, 488 nm, FITC). **B** and **C**, the groom time and bouts in 24 h of *Shank3*^-/-^:*p38α*^*AgRP-con*^ and *Shank3*^-/-^:*p38α*^*AgRP-180/182*^ mice. **D**. the total distance in 24 h of *Shank3*^-/-^:*p38α*^*AgRP-con*^ and *Shank3*^-/-^:*p38α*^*AgRP-180/182*^ mice. **E**. the activity time in 24 h of *Shank3*^-/-^:*p38α*^*AgRP-con*^ and *Shank3*^-/-^:*p38α*^*AgRP-180/182*^ mice. **F**. the first phase of the three-chamber test, the time of *Shank3*^-/-^:*p38α*^*AgRP-con*^ and *Shank3*^-/-^:*p38α*^*AgRP-180/182*^ mice in stranger1 chamber. **G**. the second phase of the three-chamber test, the time of *Shank3*^-/-^:*p38α*^*AgRP-con*^ and *Shank3*^-/-^:*p38α*^*AgRP-180/182*^ mice in stranger2 chamber. *Shank3*^-/-^:*p38α*^*AgRP-con*^ and *Shank3*^-/-^:*p38α*^*AgRP-180/182*^ male mice were subjected to Home-Cage monitoring test at the age of 8 weeks, and subjected to three-chamber test at the age of 14 weeks, 12 mice in *Shank3*^-/-^:*p38α*^*AgRP-con*^ and 19 mice in *Shank3*^-/-^:*p38α*^*AgRP-180/182*^ group. Statistical analysis: data were analyzed using unpaired two-tailed Student’s *t*-test (Prism9, GraphPad Software Inc.). Data were represented as Mean ± SD. Significance levels are indicated with *p < 0.05.

Inactivation of p38α in AgRP neurons did not alter stereotypic behavior and sociability in WT mice (Fig. S4). One possible reason is that WT mice are normal animals with few autistic-like behaviors. Thus, the improved effect on stereotypic behavior and sociability in WT mice is extremely difficult to observe. To address this issue, we generated a *Shank3*^-/-^:*p38α*^*AgRP-180/182*^ mice line, in which p38α was specifically inactivated in AgRP neurons of *Shank3*^-/-^ mice (Fig. 5A). As expected, *Shank3*^-/-^:*p38α*^*AgRP-180/182*^ mice showed a significant reduce in groom time (Fig. 5B) but not groom bouts (Fig. 5C), accompanied by unaltered total distance and activity time (Fig. 5D, E). Of note, *Shank3*^-/-^:*p38α*^*AgRP-180/182*^ mice showed a significant improvement in sociability (Fig. 5F, G). Together, our results demonstrate that the activity of p38α signaling in AgRP neurons is probably a therapeutic target for ASD.

### p38α in POMC neurons does not regulate stereotypic behavior and sociability

**Fig. 6:**
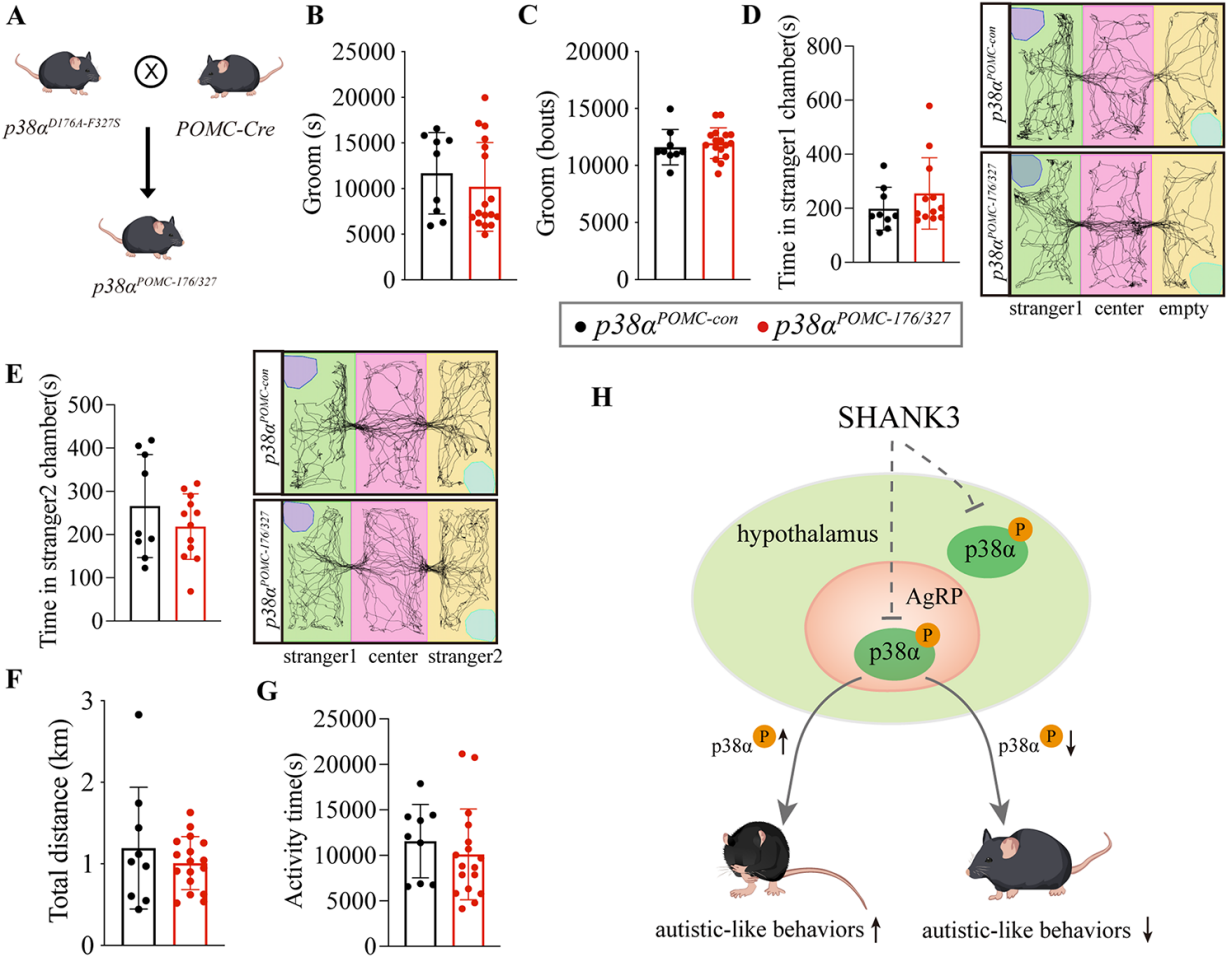
Activated p38α in POMC neurons does not affect stereotypic behavior and sociability. **A**. schematic of generating *p38α*^*POMC-176/327*^ mice. **B** and **C**, the groom time and bouts in 24 h of *p38α*^*POMC-con*^ and *p38α*^*POMC-176/327*^ mice. **D**. the first phase of the three-chamber test, the time of *p38α*^*POMC-con*^ and *p38α*^*POMC-176/327*^ mice in stranger1 chamber. **E**. the second phase of the three-chamber test, the time of *p38α*^*POMC-con*^ and *p38α*^*POMC-176/327*^ mice in stranger2 chamber. **F**. the total distance in 24 h of *p38α*^*POMC-con*^ and *p38α*^*POMC-176/327*^ mice. **G**. the activity time in 24 h of *p38α*^*POMC-con*^ and *p38α*^*POMC-176/327*^ mice. **H**. Model of p38α activity associated with stereotypic behavior and sociability. *p38α*^*POMC-con*^ and *p38α*^*POMC-176/327*^ male mice were used for experiment, 9 mice in *p38α*^*POMC-con*^ and 12 mice in *p38α*^*POMC-176/327*^ group. Mice were subjected to Home-Cage monitoring test at the age of 8–9 weeks, and subjected to three-chamber test at the age of 9–10 weeks. Statistical analysis: data were analyzed using unpaired two-tailed Student’s *t*-test (Prism9, GraphPad Software Inc.). Data were represented as Mean ± SD. Significance levels are indicated with *p < 0.05.

Since the POMC neuron is another primary neuron in ARC [13], we next explored whether POMC neurons are involved in regulating stereotypic behavior and sociability. For this reason, we generated a *p38α*^POMC*-176/327*^ mouse line (Fig. 6A) by *p38α*^*D176A-F327S*^ flox knock-in mice crossed with *POMC-Cre* mice, in which p38α is spontaneously constitutively activated in POMC neurons due to mutating in p38α residues both D176A and F327S [49]. Activated p38α in POMC neurons did not cause significant changes in stereotypic behavior (Fig. 6B, C) and social behavior (Fig. 6D, E), accompanied by unchanged total distance and activity time (Fig. 6F, G). Together, p38α in POMC and AgRP neurons play distinct roles in regulating autism-like behaviors (Fig. 6H).

## Discussion

*Shank3* is a critical gene for autism development [2]. Understanding the neuronal and molecular mechanisms of *Shank3*-related autism could help in the treatment of some subset of autism. Here, we demonstrate that deletion of *Shank3* promotes p38α signaling to elicit autistic-like behaviors. Furthermore, activated p38α in AgRP neurons elicits excessive stereotypic behavior and impaired sociability in WT mice, and inactivated p38α improves autistic-like behaviors in *Shank3*^-/-^ mice. Our results suggest that the internal SHANK3/p38α signaling pathway in AgRP neurons is an important signaling transduction cascade for autistic behaviors, as elucidated in Fig. 6H.

In this work, we unveiled an unexpected role for AgRP neurons in controlling stereotypic behavior and sociability behaviors via p38α signaling. AgRP neurons control non-feeding behaviors by affecting the medial prefrontal cortex and regulate feeding and satiety via expressing serotonin1B receptors (5-HT_1B_R) [20]. Notably, p38α in 5-HT neurons drives autistic-like phenotypes in ASD model [18, 19] and 5-HT receptors are the therapeutic target of multiple antipsychotic medications [51], suggesting that 5-HT receptors are the downstream effector of SHANK3/p38α pathway. Consistently, inhibition of MNK1/2, a downstream of p38α, restores social behavior in ASD mouse model [27]. Thus, the p38α signaling is a potential target for developing drugs for treating autism, especially for the *Shank3*-related subset population.

Inactivation of p38α in AgRP neurons did not significantly promote the groom bouts of *Shank3*^-/-^ mice (Fig. 5C), indicating that other signaling pathways are associated with the regulation of stereotypic behaviors. *Shank3* deficiency leads to abnormalities in brain structure and molecular signaling pathways, suggesting that the effects of *Shank3* deficiency are widespread, multifarious, and profound [52]. Thus, modulation of a specific molecular pathway in AgRP neurons is impossible to rescue the complete effects of *Shank3* deficiency.

Stereotypic behavior and sociability in WT mice are unchanged by the deficiency or inactivation of p38α in AgRP neurons (Figs. S1, S2, S4). There are several possible reasons for these results. First, WT mice hardly exhibit the improved effect on stereotypic behavior and sociability, as evidenced by the inactivation of p38α in AgRP neurons improves the autistic-like behaviors in *Shank3*^-/-^ mice (Fig. 5). The second possible reason is the presence of functionally redundant p38 isoforms or intracellular compensatory MAPK subfamilies similar to a previous report [9]. Consistently, our immunoblotting data showed that Erk1/2 and JNK phosphorylation levels were also upregulated in the hypothalamus of *Shank3*^-/-^ mice (Fig. 1C). Thus, we cannot rule out the possibility that Erk1/2 and JNK act as a regulator for stereotypic behavior and sociability. Indeed, Erk signaling is essential for neural development in the brain [53] and ERK2 regulates SHANK3 stability in vivo [47]. Furthermore, the JNK pathway appears to be associated with the autistic population with intellectual disability [54]. The third possibility is that multiple types of neurons are orchestrated to regulate stereotypic behavior and sociability. Indeed, ARC contains multiple types of neurons and glial cells [13, 55]. Finally, it is also possible that all or some of these reasons are orchestrated to regulate autistic-like behaviors.

### Limitations

This paper demonstrated that SHANK3 regulates the phosphorylated p38 level in the hypothalamus and inactivated p38α in AgRP neurons significantly ameliorates autistic behaviors of *Shank3*^*−/−*^ mice. However, we did not clarify the relationship between SHANK3 and p38α in AgRP neurons. Although the level of p38α phosphorylation was also elevated in the hypothalamus of BTBR mice, we did not determine whether controlling the activity of p38α in AgRP improved autistic-like behaviors in BTBR mice. Therefore, further experiments are required to identify whether the activity of p38α in AgRP neurons modulates all types of autism.

## Conclusions

Our results unveil that p38α signaling in AgRP neurons plays a critical role in the development of autism, particularly in the SHANK3 pathway mutant-related population. These findings will provide unique insights into the molecular mechanism for autistic behaviors and better therapeutic targets for autism.

### Electronic supplementary material

Below is the link to the electronic supplementary material.

Supplementary Material 1

Supplementary Material 2

Supplementary Material 3

## Data Availability

No datasets were generated or analysed during the current study.

## Abbreviations

5-HT: 5-hydroxytryptamine
5-HT1BR: serotonin1B receptors
AAV9: adeno-associated viruses 9
ARC: arcuate nucleus
AgRP: agouti-related peptide
ASD: autism spectrum disorders
Cre: cyclization recombination enzyme
CMV: cytomegalovirus (promotor)
DAPI: 4’,6-diamidino-2-phenylindole
EGFP: enhanced green fluorescent protein
FITC: fluorescein isothiocyanate
GAPDH: glyceraldehyde-3-phosphate dehydrogenase
hGH: human growth hormone gene
KEGG: Kyoto Encyclopedia of Genes and Genomes
MAPK: mitogen-activated protein kinase
MCS: multiple cloning site
MC4R: melanocortin 4 receptor
mPFC: medial prefrontal cortex
MNK1/2: MAP kinase interacting kinase
P2A: 2 A peptide
p-p38: phospho-p38
p-ERK: phospho-extracellular signal-regulated kinase
p-JNK: phospho-c-Jun NH2-terminal kinase
POMC: pro-opiomelanocortin
PI3K: phosphoinositide 3-kinase
shRNA: short hairpin ribonucleic acid
SV40: Simian vacuolating virus 40
SHANK3: SH3 and multiple ankyrin repeat domains protein 3
SERT: 5-HT transporter
TRITC: tetramethylrhodamine isothiocyanate
WT: wild-type
WPRE: woodchuck hepatitis post-transcriptional regulatory element
