# Supplementary figures and images for: Shank3 deficiency elicits autistic-like behaviors by activating p38α in hypothalamic AgRP neurons
Source: Mol Autism. 2024 Apr 3;15:14. doi: 10.1186/s13229-024-00595-4 (PMC10993499; doi:10.1186/s13229-024-00595-4)

**Figure 1D. Immunoblotting hypothalamus from wild type and *Shank3*<sup>-/-</sup> mice**

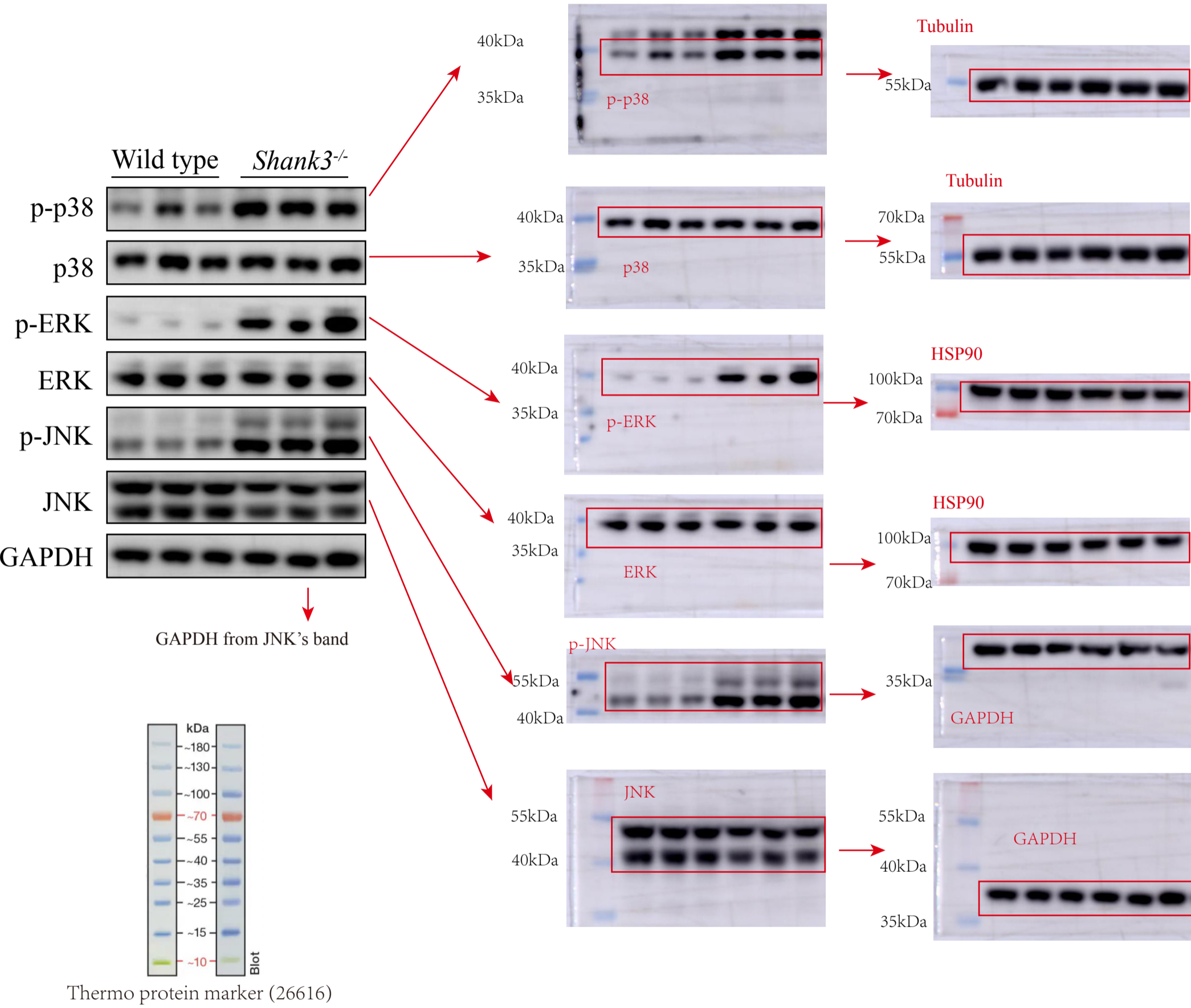

Supplement: Supplementary file 1 — Supplementary Material 1 [file 13229_2024_595_MOESM1_ESM.pdf]

**Figure 1E. Immunoblotting hypothalamus from wildtype and BTBR mice**

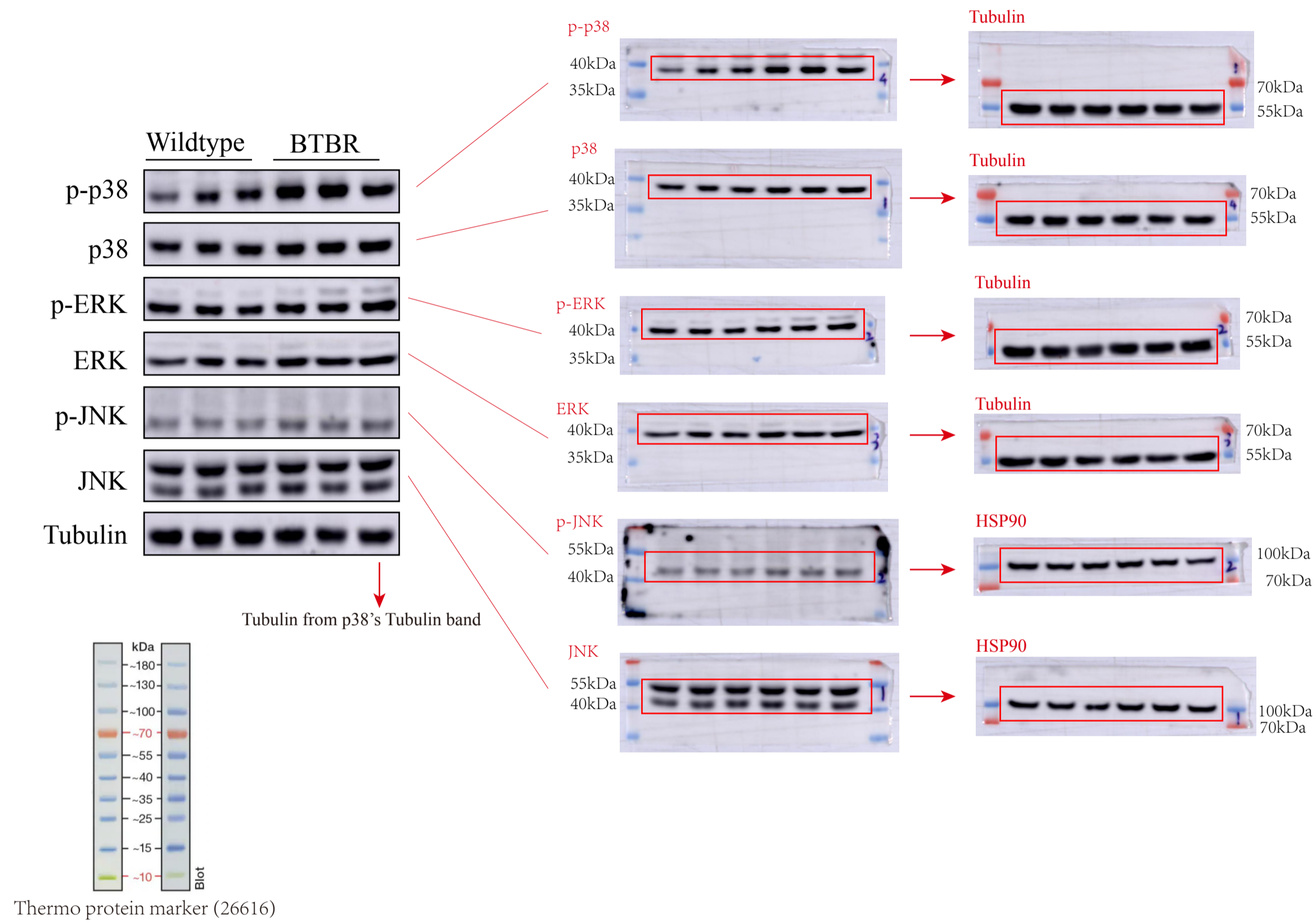

Supplement: Supplementary file 2 — Supplementary Material 2 [file 13229_2024_595_MOESM2_ESM.pdf]
