# Supplementary material for: Shank3 deficiency elicits autistic-like behaviors by activating p38α in hypothalamic AgRP neurons
Source: Mol Autism. 2024 Apr 3;15:14. doi: 10.1186/s13229-024-00595-4 (PMC10993499; doi:10.1186/s13229-024-00595-4)
Supplement: Supplementary file 3 — Supplementary Material 3 [file 13229_2024_595_MOESM3_ESM.docx]

**Supplemental Figures**


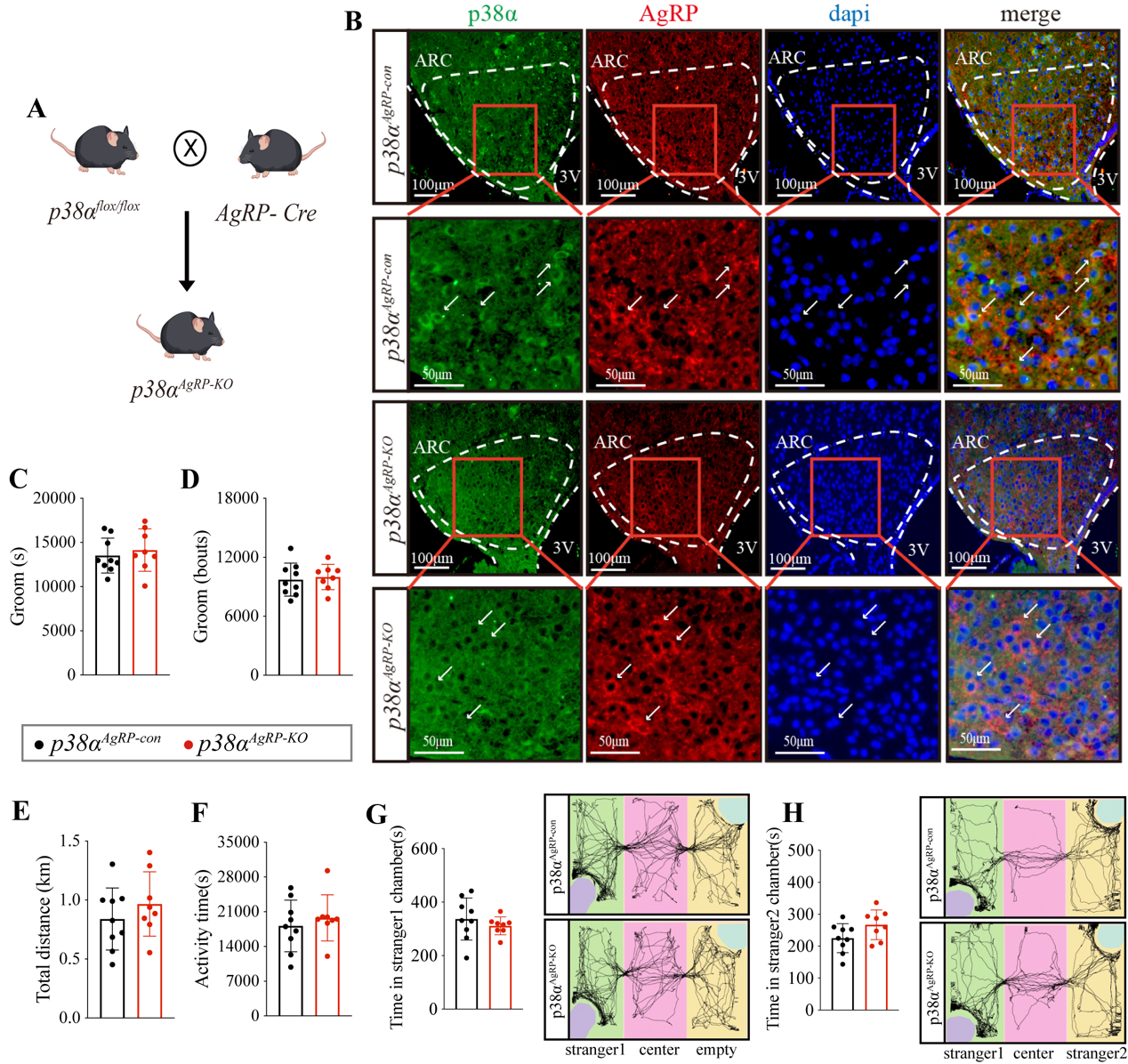


**Figure S1. Deletion of p38α in AgRP neurons does not regulate stereotypic behavior and sociability in WT mice**

**A** and **B**. Schematic of generating *p38α^AgRP-KO^* mice and immunofluorescence staining of p38α (green, 488nm, FITC), AgRP (red, 561nm, TRITC), and dapi (blue, 405nm). AgRP and p38α co-localize in *p38α^AgRP-con^* mice but not in *p38α^AgRP-KO^* mice (white arrows). **C** and **D**. The groom time and bouts in 24 hours of *p38α^AgRP-con^* and *p38α^AgRP-KO^* mice. **E**. The total distance in 24 hours of *p38α^AgRP-con^* and *p38α^AgRP-KO^* mice. **F**. The activity time in 24 hours of *p38α^AgRP-con^* and *p38α^AgRP-KO^* mice. **G**. The first phase of the three-chamber test, the time of *p38α^AgRP-con^* and *p38α^AgRP-KO^* mice in stranger1 chamber. **H**. The second phase of the three-chamber test, the time of *p38α^AgRP-con^* and *p38α^AgRP-KO^* mice in stranger2 chamber. Mice were subjected to Home-Cage monitoring test at the age of 8 weeks, and to three-chamber test at the age of 14 weeks. 9 mice for *p38α^AgRP-con^* and 8 mice for *p38α^AgRP-KO^* group. Statistical analysis: data were analyzed using unpaired two-tailed Student’s t-test (Prism9, GraphPad Software Inc.). Data represented as Mean ± SD.


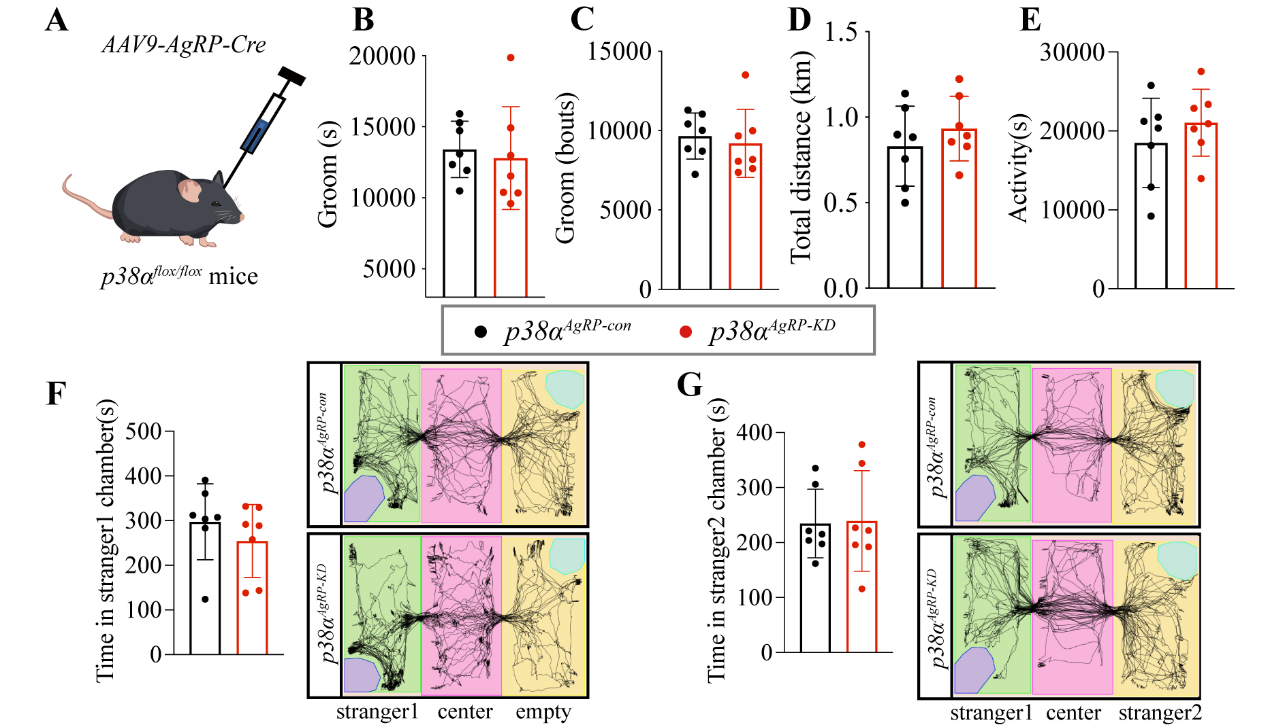


**Figure S2. Knockdown of p38α in AgRP neurons does not regulate stereotypic behavior and sociability in WT mice**

**A**. Schematic of generating *p38α^AgRP-KD^* mice (*AAV9-Agrp-Cre* mix injection to ARC of *p38α^flox/flox^* mice). **B** and **C**. The groom time and bouts in 24 hours of *p38α^AgRP-con^* and *p38α^AgRP-KD^* mice. **D**. The total distance in 24 hours of *p38α^AgRP-con^* and *p38α^AgRP-KD^* mice. **E**. The activity time in 24 hours of *p38α^AgRP-con^* and *p38α^AgRP-KD^* mice. **F**. The first phase of the three-chamber test, the time of *p38α^AgRP-con^* and *p38α^AgRP-KD^* mice in stranger1 chamber. **G**. The second phase of the three-chamber test, the time of *p38α^AgRP-con^* and *p38α^AgRP-KD^* mice in stranger2 chamber. 8-week-old wildtype male mice were injected with virus. The injection site is in the ARC area as an inclusion criterion. Total 10 injected mice in the *p38α^AgRP-con^* group, 3 mice were excluded and 7 mice were included in the experiment. Total 10 injected mice in the *p38α^AgRP-KD^* group, 3 mice were excluded and 7 mice were included in the experiment. Mice were subjected to Home-Cage monitoring test at 4 weeks after virus injection, three-chamber test was performed 5 weeks after virus injection. Statistical analysis: data were analyzed using unpaired two-tailed Student’s t-test (Prism9, GraphPad Software Inc.). Data represented as Mean ± SD.


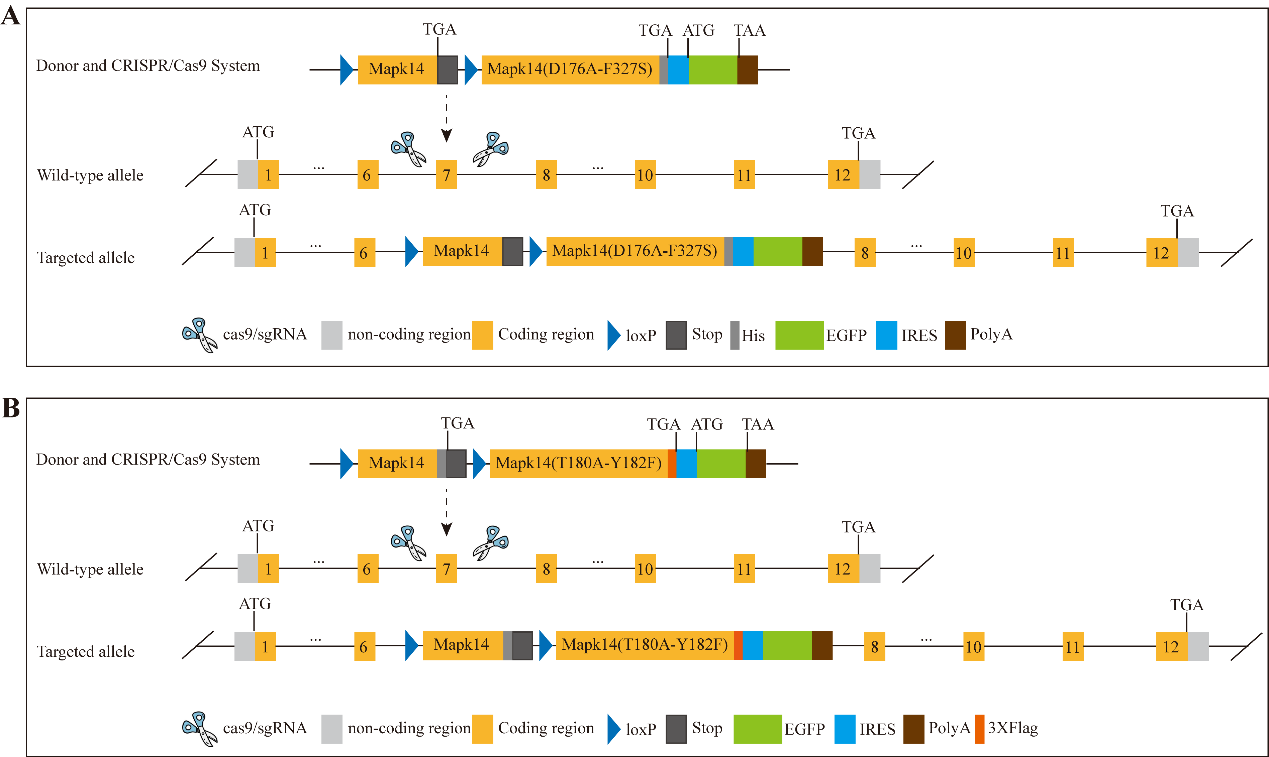


**Figure S3. Generation diagram of knock-in mice line**

**A**. Generation diagram of *p38α^AgRP-176/327^* mouse line. **B**. Generation of *p38α^AgRP-180/1827^* mouse line. sgRNA: small guide ribonucleic acid. EGFP: enhanced green fluorescent protein. IRES: internal ribozyme entry site.


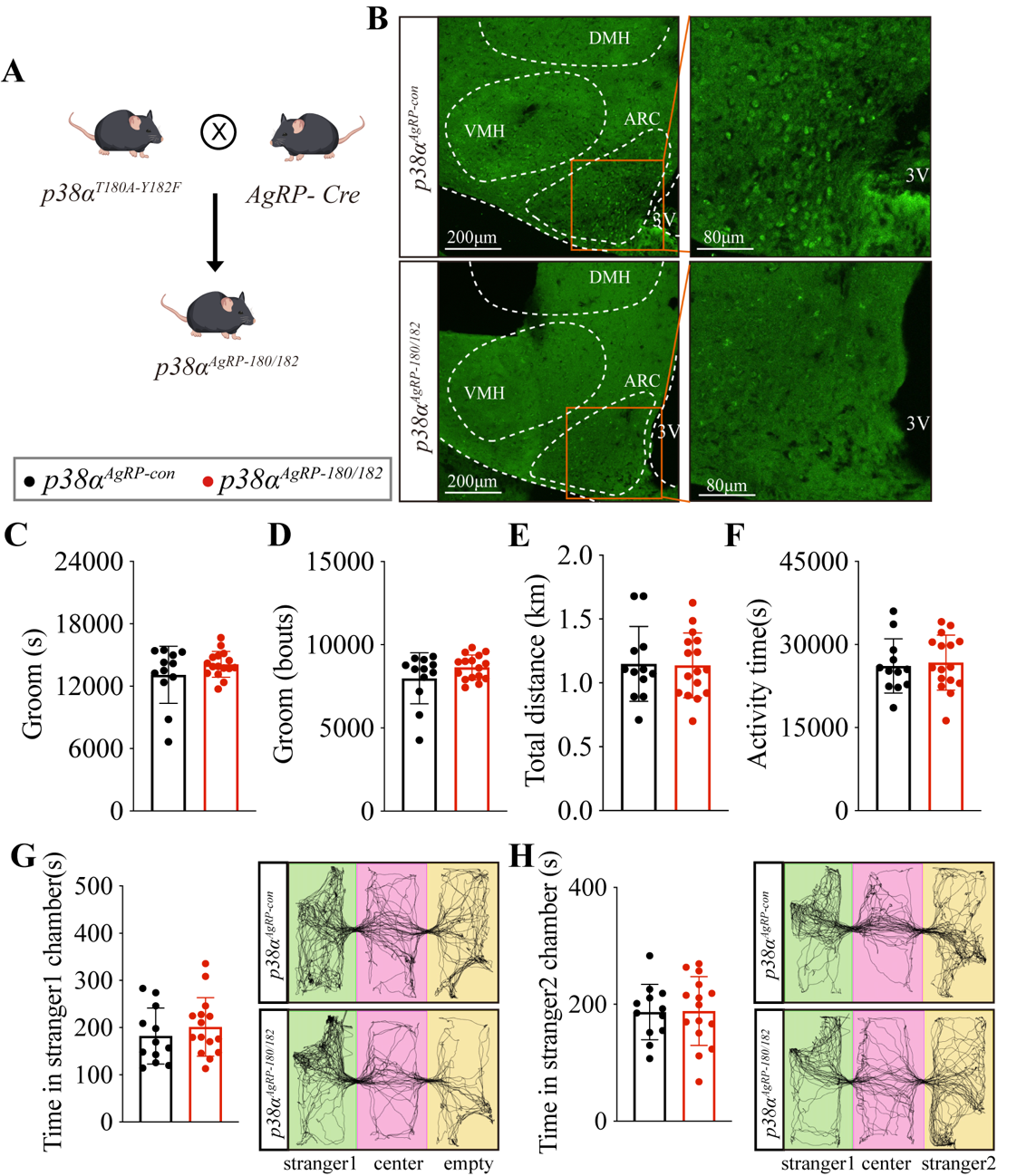


**Figure S4. Inactivate p38α in AgRP neurons does not regulate stereotypic behavior and sociability in WT mice**

**A** and **B**. Schematic of generating *p38α^AgRP-180/182^* mice and immunofluorescence staining of p-p38α (green, 488nm, FITC, 1mg/kg LPS intraperitoneal injection for 30min). **C** and **D**, The groom time and bouts in 24 hours of *p38α^AgRP-con^* and *p38α^AgRP-180/182^* mice. **E**. The total distance in 24 hours of *p38α^AgRP-con^* and *p38α^AgRP-180/182^* mice. **F**. The activity time in 24 hours of *p38α^AgRP-con^* and *p38α^AgRP-180/182^* mice. **G**. The first phase of the three-chamber test, the time of *p38α^AgRP-con^* and *p38α^AgRP-180/182^* mice in stranger1 chamber. **H**. The second phase of the three-chamber test, the time of *p38α^AgRP-con^* and *p38α^AgRP-180/182^* mice in stranger2 chamber. Mice were subjected to Home-Cage monitoring test at the age of 8 weeks and to three-chamber test at the age of 14 weeks. 12 mice for *p38α^AgRP-con^* and 16 mice for *p38α^AgRP-180/182^* group. Statistical analysis: data were analyzed using unpaired two-tailed Student’s t-test (Prism9, GraphPad Software Inc.). Data represented as Mean ± SD.
